# Supplementary material for: Complexome profiling of the Chlamydomonas psb28 mutant reveals TEF5 as an early PSII assembly factor
Source: Plant Cell. 2025 Mar 17;37(6):koaf055. doi: 10.1093/plcell/koaf055 (PMC12164590; doi:10.1093/plcell/koaf055)
Supplement: koaf055_Supplementary_Data [file koaf055_supplementary_data.zip › psb28_interactive.zip!/plots/intensity/qualityPlots/peptides/TOM20_Cre12.g534600.t1.2.html]

### Cre12.g534600.t1.2

Trivial name: TOM20  
SeqCoverage [median]: 24.90  
Mass: 21.68 kDa  
  
 

| Sequence | MissedCleavage | Mass [Da] | wtIntensity | muIntensity | Score |
| --- | --- | --- | --- | --- | --- |
| ALFGDDADREQFFEAAR | 1 | 1956.90 | 1449.47 | 0.00 | 70.78 |
| ALQNFDEAK | 0 | 1034.50 | 2513.74 | 4010.51 | 112.84 |
| ALQNFDEAKK | 1 | 1162.60 | 3343.92 | 6907.52 | 97.81 |
| EFEADNANVQALVR | 0 | 1574.77 | 4582.35 | 16072.34 | 238.92 |
| GSAPKPTAS | 1 | 814.42 | 1991.96 | 2384.05 | 111.95 |
|  |  |  | 13881.4 | 29374.4 |  |
